# Exercise in Water Provides Better Cardiac Energy Efficiency Than on Land

**DOI:** 10.3389/fcvm.2021.747841

**Published:** 2021-12-13

**Authors:** Marina Fukuie, Daisuke Hoshi, Tatsuya Hashitomi, Koichi Watanabe, Takashi Tarumi, Jun Sugawara

**Affiliations:** ^1^Graduate School of Comprehensive Human Sciences, University of Tsukuba, Tsukuba, Japan; ^2^Human Informatics and Interaction Research Institute, National Institute of Advanced Industrial Science and Technology, Tsukuba, Japan; ^3^Faculty of Health and Sport Sciences, University of Tsukuba, Tsukuba, Japan

**Keywords:** water-based exercise, subendocardial viability ratio, myocardial oxygen consumption, aortic hemodynamics, cardiovascular response

## Abstract

Although water-based exercise is one of the most recommended forms of physical activity, little information is available regarding its influence on cardiac workload and myocardial oxygen supply-to-demand. To address this question, we compared subendocardial viability ratio (SEVR, the ratio of myocardial oxygen supply-to-demand), cardiac inotropy (*via* the maximum rate of aortic pressure rise [dP/dT_max_]), and stroke volume (SV, *via* a Modelflow method) responses between water- and land-based exercise. Eleven healthy men aged 24 ± 1 years underwent mild- to moderate-intensity cycling exercise in water (WC) and on land (LC) consecutively on separate days. In WC, cardiorespiratory variables were monitored during leg cycling exercise (30, 45, and 60 rpm of cadence for 5 min each) using an immersible stationary bicycle. In LC, each participant performed a cycling exercise at the oxygen consumption (VO_2_) matched to the WC. SEVR and dP/dT_max_ were obtained by using the pulse wave analysis from peripheral arterial pressure waveforms. With increasing exercise intensity, SEVR exhibited similar progressive reductions in WC (from 211 ± 44 to 75 ± 11%) and LC (from 215 ± 34 to 78 ± 9%) (intensity effect: *P* < 0.001) without their conditional differences. WC showed higher SV at rest and a smaller increase in SV than LC (environment-intensity interaction: *P* = 0.009). The main effect of environment on SV was significant (*P* = 0.002), but that of dP/dT_max_ was not (*P* = 0.155). SV was correlated with dP/dT_max_ (*r* = 0.717, *P* < 0.001). When analysis of covariance (ANCOVA) was performed with dP/dT_max_ as a covariate, the environment effect on SV was still significant (*P* < 0.001), although environment-intensity interaction was abolished (*P* = 0.543). These results suggest that water-based exercise does not elicit unfavorable myocardial oxygen supply-to-demand balance at mild-to-moderate intensity compared with land-based exercise. Rather, water-based exercise may achieve higher SV and better myocardial energy efficiency than land-based exercise, even at the same inotropic force.

## Introduction

Aerobic exercise, such as walking, jogging, and cycling is a beneficial health strategy for non-pharmacologically preventing and treating cardiovascular disease. However, not all populations, particularly those with decreased walking ability, musculoskeletal pain and disorders, and anxiety of falling, can engage in regular exercise training. All these factors can decrease motivation and continuation of exercise. Alternatively, water-based exercise reduces a gravitational load on joints due to buoyancy, and the risk of falling is lower due to water resistance (1). Therefore, water-based exercise may be a suitable modality without pain and discomfort for populations who have difficulty exercising on land (2, 3).

Stroke volume (SV) increases with water immersion *per se* and further with water-based exercise to a greater extent than land-based exercise (4–6). In water, altered hydrostatic pressure shifts peripheral venous blood toward the heart and increases SV based on Frank-Starling's law. In contrast, the increase in central blood volume raises the cardiac volume load (preload), which determines myocardial oxygen consumption regardless of healthy or diseased conditions (7, 8). Additionally, the exercise-induced increases in heart rate (HR) and cardiac contractility may raise myocardial oxygen demand (9). Therefore, the clinicians have long been concerned that increases in central blood volume and cardiac preload may not be tolerated by patients with chronic heart failure and potentially worsen their symptoms and exercise capacity (10). With this prevailing concern as a background, Adsett et al. conducted a meta-analysis to determine the efficacy of aquatic exercise training for patients with heart failure compared with the traditional land-based exercise programs. By including eight interventional studies, they found that, in patients with stable heart failure, aquatic exercise training could improve exercise capacity, muscle strength, and quality of life similar to the land-based exercise programs (10). However, little information is yet available regarding myocardial oxygen demand and supply balance during water-based exercise in healthy individuals.

As an initial step to better understand the safety of water-based exercise, we investigated the impact of mild-to-moderate intensity, water-based exercise on myocardial oxygen demand and supply in young, healthy individuals. To address this aim, we compared the aortic time-tension and diastolic pressure-time integrals (TTI and DTI; indices of myocardial oxygen demand and supply) and subendocardial viability ratio (SEVR = DTI/TTI) (11, 12) during cycling with the matched oxygen consumption (VO_2_) in water and on land. We hypothesized that water-based exercise would be associated with higher myocardial oxygen demand because it increases central blood volume and cardiac preload, although myocardial oxygen supply-to-demand balance would be similar to that during the land-based exercise.

## Materials and Methods

### Participants

Eleven healthy men [age: 24 ± 1 years, height: 174.7 ± 4.5 cm, weight: 69.1 ± 4.9 kg, body mass index (BMI): 22.6 ± 1.1 kg · m^−2^] participated in this study. All participants were nonsmokers, not taking any medication, and had no history of cardiovascular, respiratory, neuromuscular, or musculoskeletal disorders or diseases. This study was approved by the Research Ethics Committee of the University of Tsukuba. All participants gave informed consent before participation.

### Experimental Protocol

Each participant visited the laboratory three times and had the following measurements on each day: peak oxygen consumption (VO_2peak_), water-based cycling (WC) test, and land-based cycling (LC) test. Participants were instructed to fast for a minimum of 3 h and to refrain from vigorous exercise, alcohol consumption, and caffeine intake at least 24 h before each visit. All visits were separated by at least 72 h.

#### Day 1: VO_2peak_ Measurement

VO_2peak_ was calculated by averaging the 30 s before the finish of the exercise. The test utilized a recumbent bike (Corival recumbent, Lobe, BV, Netherlands) and an expiratory gas analyzer (Aero monitor, MINATO, Osaka, Japan) to assess oxygen uptake during exercise. The workload started with 60 W and then increased by 20 W every 2 min. After reaching 160 W, the workload was increased by 10 W until the two of the following criteria are met: (1) 95% HR reserve calculated from the age-predicted maximum, (2) respiratory exchange ratio >1.2, (3) unable to maintain cycling at 60 ± 5 rpm, or (4) rate of perceived exertion (RPE) higher than 19.

For familiarization, participants practiced 15 min of cycling exercise with no-load (i.e., WC with no paddles and LC with 0 W) before each main trial of WC (*Day 2*) and LC (*Day 3*). The cadence increased by 15 rpm every 5 min in each environment to get used to cycling exercise.

#### Day 2: Water-Based Cycling

Participants wore swim shorts and rested in the seated position on land for 5 min. Then, they moved and sat on a stationary semi-recumbent bicycle (Hydrorecline, H3Oz company, Italy) in water (water temperature: 31–32°C, xiphoid level) for 4 min, and started cycling exercise. The cycling exercise consisted of three stages at which participants cycled at 30, 45, and 60 rpm incrementally for 5 min each. Each participant kept the cadence according to the metronome sound. To increase pedaling resistance, three paddles (7 cm × 22 cm × 0.5 cm, made of plastic) were attached to the bottom bracket axle. During the exercise test, the cardiorespiratory and hemodynamic variables were monitored. VO_2_ recorded at each stage of cycling in water was used to match VO_2_ during land-based cycling.

#### Day 3: Land-Based Cycling

Participants rested in the seated position on land (room temperature: 25–26°C) for 5 min and rested for another 4 min to match with the WC. After resting, participants conducted 15 min of cycling exercise using a recumbent bicycle (Corival recumbent, Lode BV, Netherlands). Each participant performed three stages of a 5-min cycling exercise. They maintained the cadence of 60 rpm while the load was manually adjusted to match VO_2_ to the corresponding stages of the WC. The cardiorespiratory and hemodynamic variables were recorded continuously and reported, and the data were calculated by averaging the last continuous and stable 1 min of each stage.

### Measurements

#### Body Temperature and Perceived Exercise Intensity

At 2 and 4 min of each stage, the RPE scale by using the Borg scale (13) and tympanic temperature during training was listened to and measured.

#### Systemic Hemodynamics

Digital arterial pressure waveform was continuously recorded at the right middle finger by a non-invasive blood pressure monitor (Human NIBP Nano System, AD Instrument, Colorado Springs, CO, USA) and stored on a computer using a data acquisition system (PowerLab, AD Instrument, Colorado Springs, CO, USA) at the sampling rate of 1 kHz for offline analysis. Participants kept their right hand at the heart level (on the side table outside the bathtub during WC) throughout resting and exercise testing. The pressure values provided by this type of device do not significantly differ from those taken directly from the radial artery under various physiological conditions (14). There is good agreement in the evaluation of beat-to-beat variations (15). Beat-to-beat HR and systolic, diastolic, and mean arterial pressures (SBP, DBP, and MAP) and pulse pressure (PP) were calculated from pulse waveforms. SV, cardiac output (CO), and systemic vascular resistance (SVR = MAP/CO) were estimated by the Modelflow-based add-on program for the data acquisition system. The validity of this method has been established in a variety of conditions that include exercise (16, 17).

#### Aortic Hemodynamics

**Figure 1:**
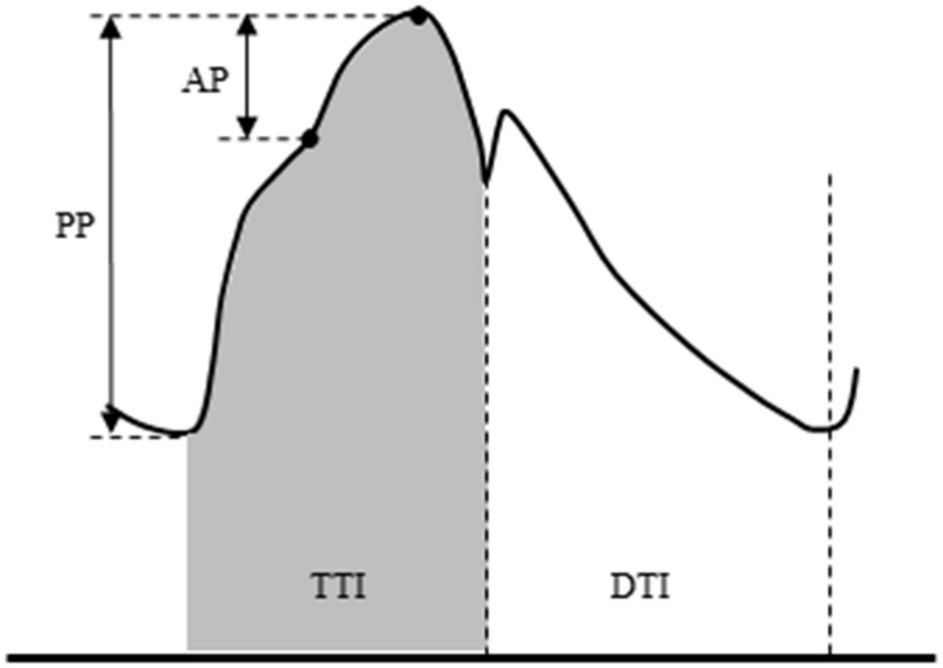
Schema of aortic pulse wave analysis. Augmentation pressure (AP) is the difference between the first systolic peak (or shoulder) and the second systolic peak pressure. Augmentation index (AIx) is defined as the AP as a percentage of pulse pressure. The dicrotic notch (DN) represents the closure of the aortic valve and is used to calculate ejection duration. Aortic tension-time index (TTI) and diastolic pressure-time index (DTI) are areas under the aortic systolic and diastolic pressure-time curve (both measured from 0 mmHg) and reflect the myocardial oxygen demand and the blood supply to the heart, respectively. The subendocardial viability ratio (SEVR) is the ratio of DTI to TTI.

Aortic blood pressure waveforms were synthesized from digital artery pressure waveforms *via* a generalized transfer function (SphygmoCor, AtCor Medical, Sydney, Australia) as we previously reported (18–20). We (20) and another group (21) have confirmed the validity of applying this technique during exercise. Aortic pressure was calibrated with an oscillometric SBP and DBP of the brachial artery. A typical aortic pressure waveform is depicted in Figure 1. Aortic augmentation pressure (AP) was defined as the difference between the first systolic peak (or shoulder) and the second systolic peak pressure. Aortic augmentation index (AIx) was calculated as the percentage ratio of aortic AP to aortic PP. The companion matric of AIx (AIxC) being the hypotenuse of the triangle formed by AP and PP (*via* the Pythagorean theorem) was also calculated (22). The aortic compliance index was calculated by dividing SV by aortic PP (23). Ejection duration was defined as the time from the systolic foot to the aortic dicrotic notch. The diastolic duration was calculated as the time of the cardiac cycle minus ejection duration. Areas under the aortic systolic and diastolic pressure-time curve (both measured from 0 mmHg) were calculated as TTI and DTI for estimates of myocardial oxygen consumption (e.g., demand) and perfusion (e.g., supply) (24–26). SEVR was then calculated as the ratio of DTI/TTI × 100 (%). This index is related to the subendocardial-to-subepicardial blood flow ratio and analogous to subendocardial perfusion (12). The dP/dT_max_, the maximal rate of pressure rise in the upstroke portion of the aortic waveform, was calculated to estimate the left ventricular inotropic state (27).

### Statistical Analysis

Data were calculated by averaging the last 1 min of each stage. For the main effects of environment and exercise intensity and their interaction effect, a two-way repeated ANOVA was applied. In the case of a significant interaction effect, a *post-hoc* test [Fisher's least significant difference (LSD)] was performed. Pearson's product-moment correlation was used to determine the relationship between variables of interest. Analysis of covariance (ANCOVA), using dP/dT_max_ or HR as a covariate, was used to analyze the effects of environment and exercise intensity on SV and AIx, respectively. Exercise-induced changes in AIxC were compared between the WC and LC, respectively, by using the paired *t*-test. All data are reported as mean ± SD. Statistical significance was *a priori* defined as a *P* < 0.05.

## Results

The VO_2peak_ and the peak workload were 36.4 ± 4.3 ml · kg^−1^ · min^−1^ and 214 ± 17 W, respectively. Absolute and relative VO_2_ progressively increased in WC and LC with no significant intensity-environment interaction effect (Table 1), indicating that VO_2_ was matched in both conditions at each intensity. The body temperature response to exercise showed no difference between the WC and LC. RPE response showed no significant difference between environments (*P* = 0.844) despite a significant interaction (*P* = 0.012).

**Table 1:** Change in the body temperature and exercise intensity.

		**Rest**	**Stage 1**	**Stage 2**	**Stage 3**	***P*** **-value**		
		**Mean ± SD**	**Mean ± SD**	**Mean ± SD**	**Mean ± SD**	**Intensity**	**Environment**	**Interaction**
Cadence, rpm	WC	0	30	45	60			
	LC	0	60	60	60			
Workload, watt	WC	–	–	–	–			
	LC	0 ± 0	13 ± 4	45 ± 11	106 ± 17			
Body temperature, °C	WC	35.3 ± 0.5	35.3 ± 0.6	35.4 ± 0.6	35.4 ± 0.5	0.002	0.973	0.801
	LC	35.3 ± 0.4	35.4 ± 0.4	35.4 ± 0.4	35.4 ± 0.4			
VO_2_, ml · kg^−1^ · min^−1^	WC	3.3 ± 0.5	5.6 ± 0.8	9.0 ± 0.8	16.8 ± 1.8	<0.001	0.165	0.233
	LC	3.2 ± 0.4	6.0 ± 0.8	9.4 ± 0.9	17.0 ± 2.3			
%VO_2peak_ %	WC	9.2 ± 1.8	15.7 ± 3.4	25.0 ± 4.3	46.9 ± 8.0	<0.001	0.187	0.238
	LC	8.8 ± 1.4	16.7 ± 3.3	26.3 ± 4.7	47.5 ± 9.8			
RPE, a.u.	WC	6.5 ± 0.9	7.3 ± 1.1^a^	9.3 ± 2^ab^	12.4 ± 1.5^abc^	<0.001	0.844	0.012
	LC	6.4 ± 0.9	7.7 ± 1.3^a^	9.3 ± 2^ab^	11.6 ± 1.6^abc^			

*Data are mean ± SD. WC, cycling in water condition; LC, cycling on land condition; VO_2_, oxygen consumption; %VO_2peak_ the percent peak oxygen consumption measured by a land-based incremental cycling test; RPE, rate of perceived exertion. a, b, and c indicate significant differences from rest, stage 1, and stage 2 in the same environment, respectively*.

**Figure 2:**
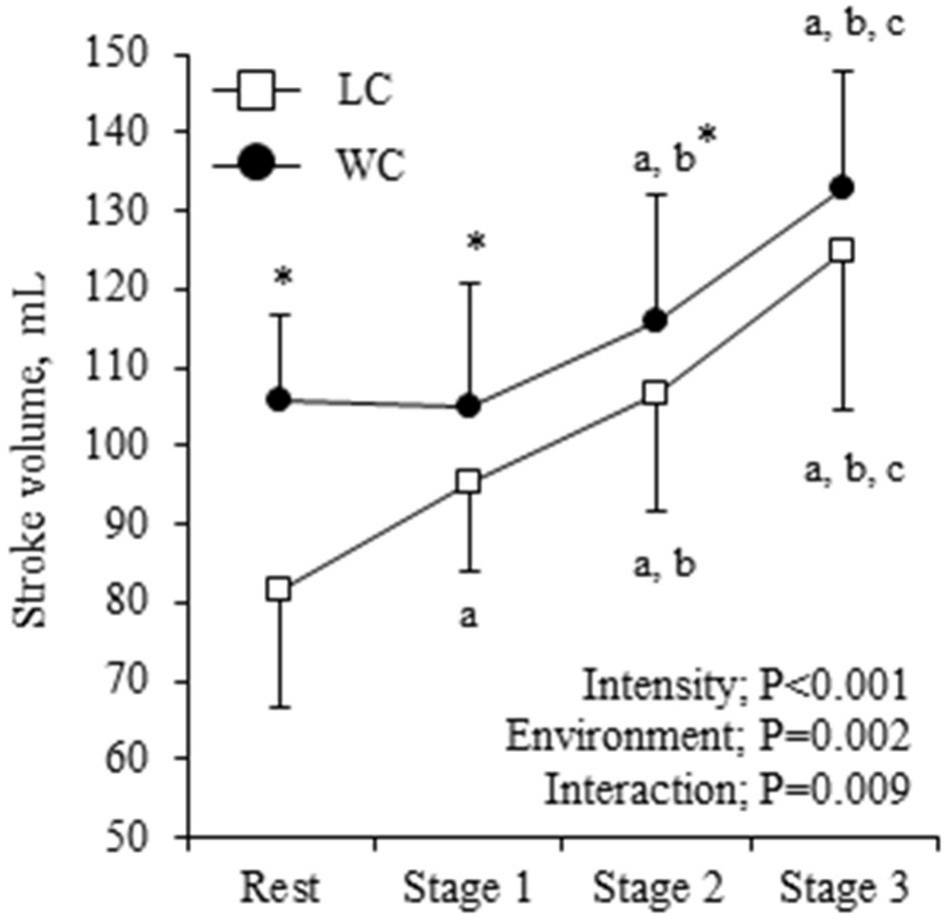
Stroke volume (SV) response at rest and during exercise. Symbols and error bars are mean and SD. The circle and square indicate water-based and land-based exercise, respectively. The *P*-values for the two-way repeated ANOVA data are presented (environment, intensity, and interaction). ^a,b,c^show significant differences from rest, stage 1, and stage 2 of the same environment, respectively. ^*^indicates a significant difference from land at the same stage.

Systemic hemodynamic variables at rest and during exercise are shown in Table 2. WC showed significantly higher CO and lower HR than LC (environment effect: *P* = 0.021 and 0.012, respectively), and they progressively increased as exercise intensity increased (intensity effect: *P* < 0.001 for both). Environment-intensity interactions were not significant for HR and CO. SV showed a significant environment-intensity interaction (*P* = 0.009, Figure 2). The *post-hoc* test revealed that WC was significantly higher than LC at rest to stage 2. Additionally, SVR showed a significant environment-intensity interaction (*P* = 0.001); significantly lower in WC than LC at rest, stage 1, and stage 2. Brachial SBP and DBP were significantly lower in WC than LC (environment effect: *P* = 0.001 for both). With the increase in intensity, SBP gradually increased (intensity effect: *P* = 0.011), and DBP was not affected by the intensity (*P* = 0.186).

**Table 2:** Systemic hemodynamics at rest and during exercise.

		**Rest**	**Stage 1**	**Stage 2**	**Stage 3**	***P*** **-value**		
		**Mean ± SD**	**Mean ± SD**	**Mean ± SD**	**Mean ± SD**	**Intensity**	**Environment**	**Interaction**
HR, bpm	WC	55 ± 9	72 ± 9	82 ± 9	109 ± 15	<0.001	0.012	0.689
	LC	63 ± 10	75 ± 13	86 ± 13	113 ± 17			
CO, L · min^−1^	WC	6 ± 0.9	7.3 ± 1.3	9.7 ± 1.6	14.6 ± 2	<0.001	0.021	0.366
	LC	5.2 ± 1.1	6.9 ± 1.2	9.1 ± 1.3	13.9 ± 1.8			
SVR, mmHg · min^−1^ · L^−1^	WC	15 ± 3^*^	12 ± 3^a^^*^	9 ± 3^ab^^*^	7 ± 1^abc^	<0.001	<0.001	0.001
	LC	19 ± 3	15 ± 2^a^	11 ± 2^ab^	7 ± 1^abc^			
SBP, mmHg	WC	112 ± 8^*^	112 ± 9^*^	117 ± 9^ab^^*^	130 ± 11^abc^^*^	0.011	0.001	0.001
	LC	119 ± 8	123 ± 12	129 ± 10^a^	145 ± 13^abc^			
DBP, mmHg	WC	69 ± 8	67 ± 7	67 ± 6	67 ± 7	0.186	0.001	0.419
	LC	82 ± 6	78 ± 8	76 ± 9	73 ± 13			
PP, mmHg	WC	43 ± 9^*^	44 ± 10	50 ± 9^ab^	64 ± 10^abc^^*^	<0.001	0.532	0.015
	LC	37 ± 6	45 ± 9^a^	53 ± 8^ab^	72 ± 10^abc^			

*Data are mean ± SD. HR, heart rate; CO, cardiac output; SVR, systemic vascular resistance; SBP, systolic blood pressure; DBP, diastolic blood pressure; PP, pulse pressure. a, b, and c indicate significant differences from rest, stage 1, and stage 2 in the same environment, respectively*.

* *indicates significant difference vs. LC at same exercise intensity*.

**Figure 3:**
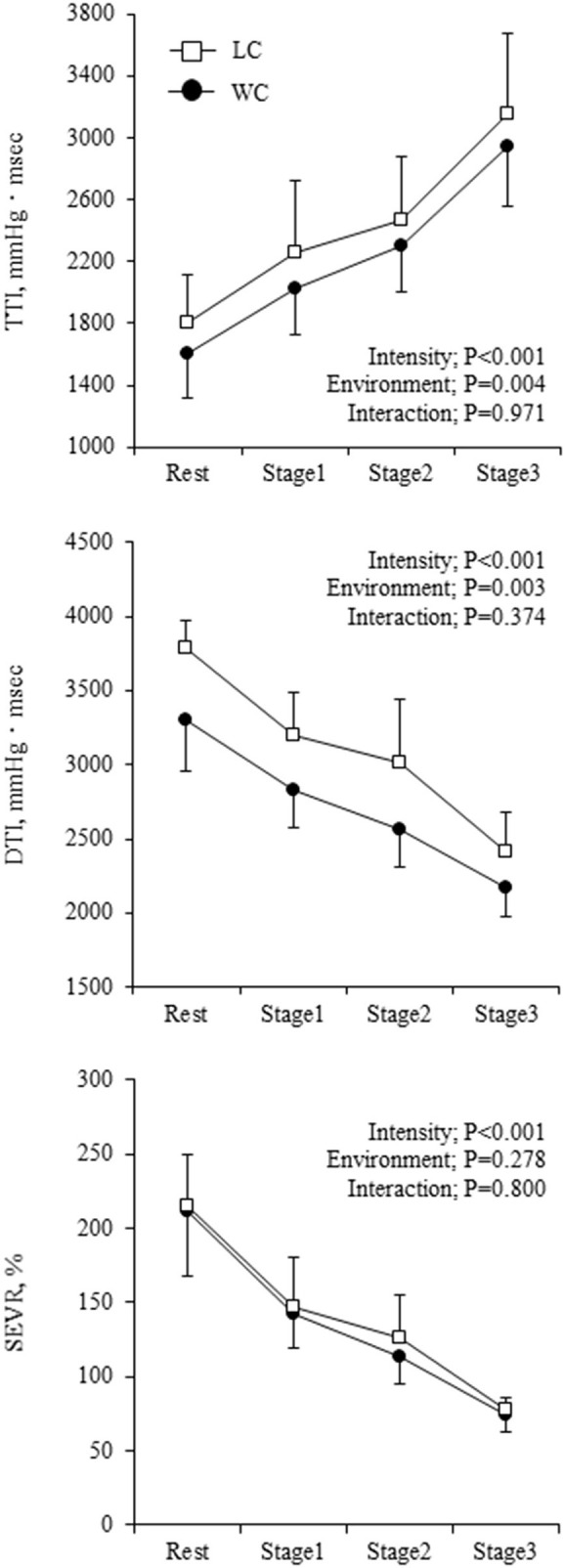
Myocardial oxygen supply-to-demand balance indicators at rest and during exercise. Symbols and error bars are mean and SD. The circle and square indicate water-based and land-based exercise, respectively. The *P*-values for the two-way repeated ANOVA data are presented (intensity, environment, and interaction).

Aortic hemodynamic variables at rest and during exercise are shown in Table 3. Aortic SBP was significantly lower in WC than LC (environment effect: *P* = 0.002) and gradually increased (intensity effect: *P* = 0.085). Aortic PP showed a significant environment-intensity interaction (*P* = 0.006); higher at the baseline (Fisher's LSD: *P* = 0.002) and lower at stage 3 (Fisher's LSD: *P* = 0.023) in WC. AP was higher in WC than LC (environment effect: *P* = 0.004) and progressively decreased with increasing intensity (*P* < 0.001). Ejection duration was longer (environment effect: *P* = 0.005), and diastolic duration tended to be longer (environment effect: *P* = 0.058) in WC than in LC. These were shortened as the intensity increased (*P* = 0.003 and *P* < 0.001, respectively). The aortic compliance index was higher in WC than in LC (environment effect: *P* = 0.042), but the effect of exercise intensity (*P* = 0.667) and the environment-intensity interaction (*P* = 0.236) on aortic compliance index was not significant. TTI and DTI were lower in WC than LC (environment effect: *P* = 0.004 and *P* = 0.003, respectively, Figure 3). TTI increased and DTI decreased with increasing exercise intensity (intensity effect: *P* < 0.001 for both). SEVR exhibited similar progressive reductions in WC (from 211 ± 44 to 75 ± 11) and LC (from 215 ± 34 to 78 ± 9; environment-intensity interaction: *P* = 0.800).

**Table 3:** Aortic hemodynamics at rest and during exercise.

		**Rest**	**Stage 1**	**Stage 2**	**Stage 3**	***P*** **-value**		
		**Mean ± SD**	**Mean ± SD**	**Mean ± SD**	**Mean ± SD**	**Intensity**	**Environment**	**Interaction**
Aortic SBP, mmHg	WC	97 ± 8	96 ± 8	97 ± 8	104 ± 9	0.085	0.002	0.718
	LC	105 ± 7	105 ± 10	107 ± 10	115 ± 12			
Aortic PP, mmHg	WC	28 ± 5^*^	27 ± 6	29 ± 4	35 ± 6^ab^^*^	<0.001	0.488	0.006
	LC	22 ± 4	26 ± 5^a^	30 ± 5^ab^	39 ± 5^abc^			
Aortic compliance index, ml • mmHg^−1^	WC	3.88 ± 0.60	3.94 ± 0.73	4.07 ± 0.73	3.91 ± 0.75	0.667	0.042	0.236
	LC	3.72 ± 0.56	3.79 ± 0.75	3.59 ± 0.68	3.24 ± 0.34			
AP, mmHg	WC	2.8 ± 1.9	2.1 ± 3.3	0.2 ± 1.9	−1.9 ± 3.9	<0.001	0.004	0.111
	LC	0.6 ± 2.7	−0.4 ± 2.5	−0.6 ± 3.3	−6.6 ± 3.4			
Cardiac period, ms	WC	1,119 ± 178	850 ± 111	740 ± 80	558 ± 68	<0.001	0.011	0.331
	LC	981 ± 154	823 ± 137	712 ± 107	540 ± 72			
Diastolic duration, ms	WC	796 ± 175	533 ± 105	427 ± 73	273 ± 52	<0.001	0.058	0.277
	LC	692 ± 140	518 ± 126	422 ± 87	267 ± 48			
Ejection duration, ms	WC	323 ± 9^*^	318 ± 13^a^	313 ± 16^a^	286 ± 19^abc^^*^	0.003	0.005	0.002
	LC	289 ± 18	305 ± 18^a^	290 ± 40	273 ± 28^abc^			

*Data are mean ± SD. WC, cycling in water condition; LC, cycling on land condition; SBP, systolic blood pressure; PP, pulse pressure; SV, stroke volume; AP, augmentation pressure. a, b, and c indicate significant differences from rest, stage 1, and stage 2 in the same environment, respectively*.

* *indicates significant difference vs. LC at same exercise intensity*.

**Figure 4:**
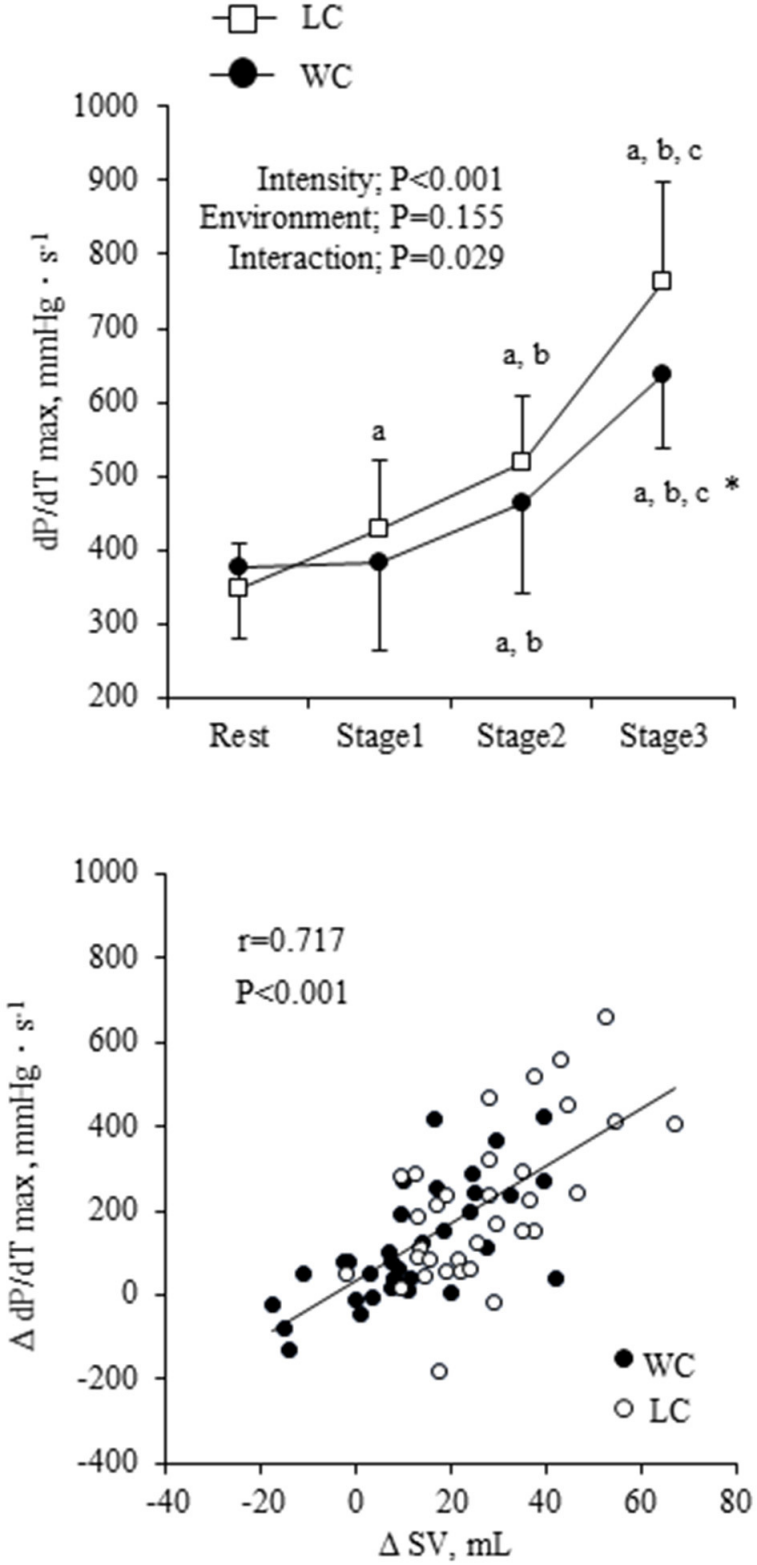
The maximum rate of aortic pressure rise (dP/dT_max_) response at rest and during exercise and scatter plot of change in SV in responses to changes in dP/dT_max_. Filled circles indicate the change in WC condition, and open squares and circles indicate the change in LC condition. ^a,b,c^show significant differences from rest, stage 1, and stage 2 of the same environment, respectively. ^*^indicates a significant difference from land at the same stage.

The dP/dT_max_ showed a significant environment-intensity interaction (*P* = 0.029) such that it gradually increased with exercise intensity and was higher in LC than WC at the highest intensity (Fisher's LSD: *P* = 0.007) (Figure 4). Change in dP/dT_max_ correlated strongly with change in SV (*r* = 0.717, *P* < 0.001). When ANCOVA was performed in SV with dP/dT_max_ as a covariate, environment-intensity interaction was abolished (*P* = 0.543), but the environment effect on SV was still significant (*P* < 0.001).

**Figure 5:**
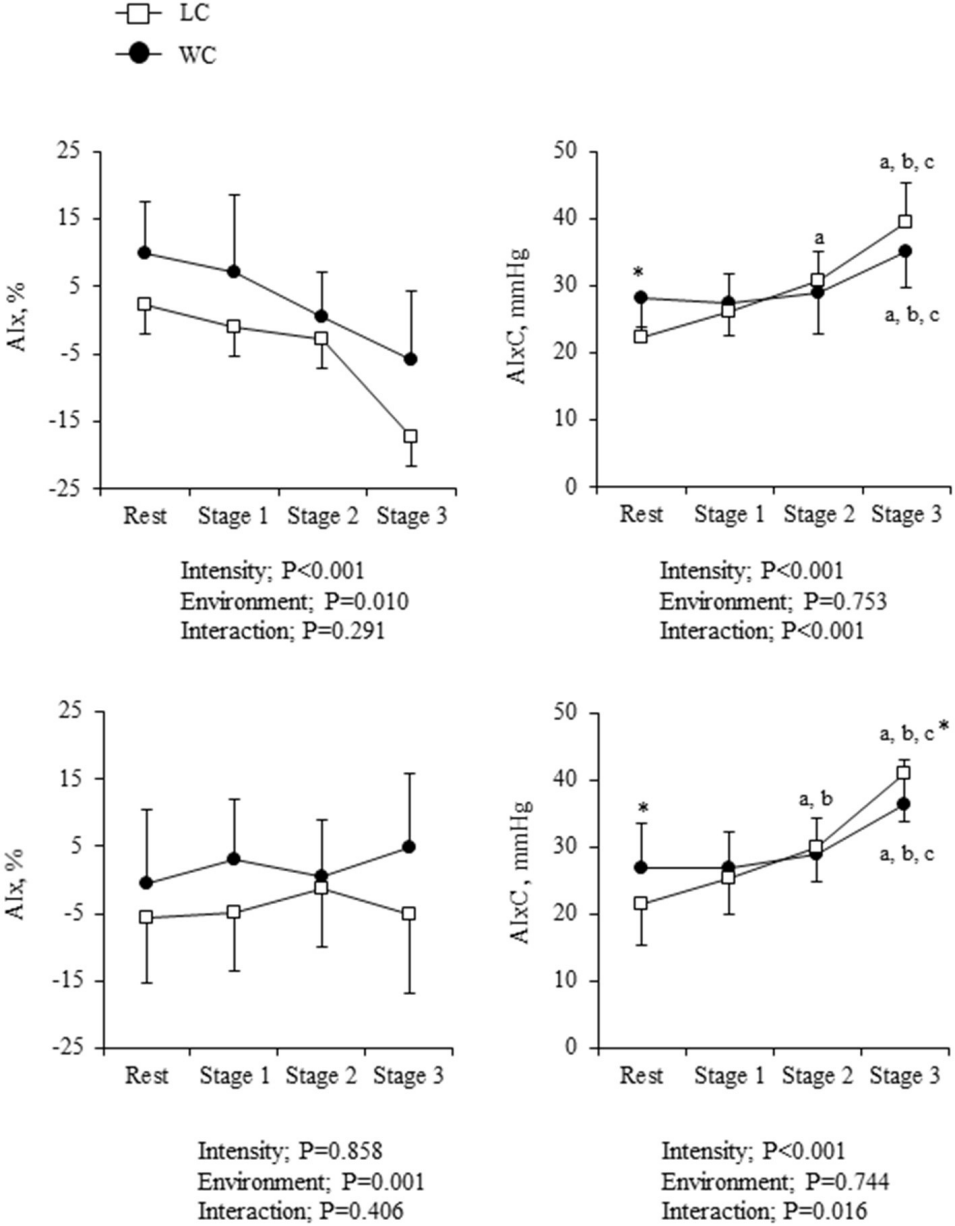
Augmentation index (AIx) and the companion metrics of AIx (AIxC) at rest and during exercise: absolute values (*top*) and heart rate (HR)-corrected values *via* analysis of covariance (ANCOVA) (*bottom*). Symbols and error bars are mean and SD. The circle and square indicate water-based and land-based exercise, respectively. ^a,b,c^show significant differences from rest, stage 1, and stage 2 of the same environment, respectively. ^*^indicates a significant difference from land at the same stage.

As shown in Figure 5, AIx was higher in WC than LC (environment effect: *P* = 0.010) and progressively decreased with increasing intensity (intensity effect: *P* < 0.001). Significant environment-intensity interaction was not found (interaction effect: *P* = 0.291). On the other hand, AIxC showed significant environment-intensity interactions (*P* < 0.001); significant differences at the rest (Fisher's LSD: *P* = 0.034) were disappeared during the exercise. The increases in AIxC from the rest to stage 3 were significantly smaller in WC than in LC (6.8 ± 7.8 vs. 16.9 ± 3.8 mmHg, *P* < 0.001). When ANCOVA was performed using HR as the covariate, the effect of environment on AIx remained significant (environment effect: *P* = 0.001), but the effect of exercise intensity was no longer significant (intensity effect: *P* = 0.858). On the other hand, the environment-intensity interaction on AIxC remained significant (interaction effect: *P* = 0.016) when HR was taken into account. The increases in AIxC from the rest to stage 3 were significantly smaller in WC than in LC (9.5 ± 3.4 vs. 19.5 ± 3.3 mmHg, *P* = 0.042).

## Discussion

The main findings of the study are as follows. First, both water- and land-based cycling at mild-to-moderate intensity induced similar reductions in the myocardial oxygen supply-to-demand ratio. Second, compared with the land condition, SV was significantly higher during water-based exercise even after dP/dT_max_ was considered using ANCOVA. These results suggest that water-based exercise does not elicit unfavorable myocardial oxygen supply-to-demand balance at mild-to-moderate intensity compared with land-based exercise. In addition, water-based exercise may be more energy-efficient for the heart because it can generate greater SV, even when the cardiac inotropy is the same as that of land-based exercise.

Water immersion may increase preload and myocardial oxygen demand. Increased hydrostatic pressure can shift peripheral venous blood toward the heart during water immersion and increase venous return. The cardiac volume loading and increased ventricular lumen radius and internal pressure may increase the myocardial oxygen demand (28). Conversely, in the present study, TTI was significantly lower in WC despite a longer ejection duration than LC. These results suggest that lower TTI was mainly due to the lower aortic SBP. Earlier studies indicated that thermoneutral head-out water immersion decreased peripheral SBP, which may be related to lowered sympathetic activity and arterial tone (29, 30). Similarly, Ueno et al. (31) demonstrated lower SVR during thermoneutral head-out water immersion in young, healthy men, though the difference did not reach statistical significance. In the current study, we observed significantly lower SVR in WC. On the other hand, since head-out cold water (~14–27°C) immersion raises SBP (29, 32, 33), TTI might be increased (33).

To the best of our knowledge, this is the first study to determine SEVR in responses to water-based exercise. In contrast to the elevation of TTI, DTI decreased with increasing exercise intensity in both conditions. The decreased DTI would be mainly due to the shortened diastolic duration because SEVR depends on the ratio of diastolic to systolic time (34). Sharman et al. examined aortic hemodynamics, such as myocardial oxygen demand, and supply during land-based cycling at 50%−80%VO_2_max in healthy young adults. SEVR decreased with increasing exercise intensity approximately from 200% at baseline to 65% during exercise at 80%VO_2_max in participants without critical cardiac symptoms (35). In the present study, SEVR exhibited similar progressive reductions in WC (from 211 ± 44 to 75 ± 11%) and LC (from 215 ± 34 to 78 ± 9%). Taken together, contrary to the conventional hypothesis that water-based exercise increases myocardial oxygen consumption and reduces cardiac work efficiency (36), such unfavorable responses were not observed during WC in this study.

It should be emphasized that the exercise-induced increase in SV was significantly smaller in WC than LC due presumably to the higher baseline level of SV. It is considered that water immersion increases central blood volume and cardiac inflow, which increases SV through the Frank-Starling mechanism that is characterized by a non-linear relation between end-diastolic volume and SV (37). Interestingly, the increase in dP/dT_max_ with exercise was smaller in WC compared with LC, and there was a strong correlation between the changes in dP/dT_max_ and SV. Besides, the absolute value of SV was significantly higher in WC than LC after dP/dT_max_ was considered. The dP/dT_max_ indicates the inotropic state of the heart during isovolumic systole which is independent of preload and afterload (27). According to the Frank-Starling mechanism, immersion-induced increases in cardiac inflow and preload seem to enhance cardiac contractility, whereas the increase in SV was smaller in WC. Ultimately, a higher cardiac inotropic effect might not be required during water-based exercise.

The aortic pressure waveform is composed of a forward traveling wave generated by left ventricular ejection and a reflected wave (e.g., AP) returning from the periphery. The earlier return of undamped reflected wave during late systole increases afterload while reducing aortic pressure during diastole, which together augments aortic pulsatile hemodynamics (38). However, in this study, many subjects exhibited negative values of AP during exercise due presumably to the delayed return of reflected wave in diastole. These observations suggest a negligible impact of wave reflection on pulsatile hemodynamics.

The arterial load would increase during progressive exercise, but we indeed observed a gradual reduction in AIx. Even after the potential effect of increased HR was adjusted using ANCOVA, AIx did not show a significant increase. This is related to increased PP (a denominator of AIx) during exercise and suggests a misinterpretation of physiological data using such dimensionless ratio-based metrics. The ratio variable, such as AIx calculated from AP and PP may lose important information underlying its physiology (22). With these rationales, this study presented AIxC, which is calculated from the hypotenuse of the AP–PP relationship based on the Pythagorean theorem (22).

Interestingly, AIxC showed a significant environment-intensity interaction; the exercise-induced increases were significantly smaller in WC than in LC (*P* = 0.042) when the effect of increased HR was adjusted. In addition, the aortic compliance index was significantly higher in WC than LC. These results suggest that the rise in arterial afterload with exercise is smaller in water than on land. The Pythagorean theorem-based transformations could provide better insight into underlying physiology. For a better understanding of physiological impact, further study is required in the field of in-water exercise.

### Study Limitations

Several methodological considerations should be mentioned. First, we studied only apparently healthy young men. To improve the generalizability and our understanding of cardiovascular physiology during water exercise, further studies are needed on other populations, such as young women, older individuals, and people with increased cardiovascular disease risk. Second, the established generalized transfer function (i.e., aorta-radial artery) was applied on finger arterial pressure waveforms measured by photoplethysmography to compute aortic blood pressure. The validity of using the general transfer function (GTF) on finger arterial pressure waveform for estimating the aortic hemodynamics was confirmed by our earlier study (18, 19). Third, semi-recumbent leg cycling exercise is not a typical exercise mode in water. However, it was suitable to administer our exercise protocols in water and on land. Notably, compared with aquatic exercise in standing posture, the impact of hydraulic pressure on the systemic and aortic hemodynamics might be smaller. Further study using other exercise modes and different underwater environments (e.g., water depth, temperature, and flow) is required. Finally, to match oxygen consumption during exercise in WC and LC, we could not conduct two protocols in random order. However, we had more than 1 week of wash-out to minimize systematic error derived by fatigue and learning effects.

## Data Availability Statement

The original contributions presented in the study are included in the article/supplementary material, further inquiries can be directed to the corresponding author/s.

## Ethics Statement

The studies involving human participants were reviewed and approved by Research Ethics Committee of the University of Tsukuba. The patients/participants provided their written informed consent to participate in this study.

## Author Contributions

JS, TT, MF, and DH decided on the conception and design of the research. MF, DH, and TH performed the experiments, analyzed the data, and interpreted the results of the experiments. MF, TT, and JS drafted the manuscript. MF and JS papered figures. All authors edited and revised the manuscript and read and approved the final manuscript.

## Funding

This work was supported by the Japanese Society for the Promotion of Science (20K11303, KW).

## Conflict of Interest

The authors declare that the research was conducted in the absence of any commercial or financial relationships that could be construed as a potential conflict of interest. The handling editor declared a past co-authorship with one of the authors TT.

## Publisher's Note

All claims expressed in this article are solely those of the authors and do not necessarily represent those of their affiliated organizations, or those of the publisher, the editors and the reviewers. Any product that may be evaluated in this article, or claim that may be made by its manufacturer, is not guaranteed or endorsed by the publisher.
